# Supplementary material for: Orthostatic hypotension in stroke/TIA patients: Association with new events and the effect of the NAILED intervention
Source: PLoS One. 2024 Feb 23;19(2):e0298435. doi: 10.1371/journal.pone.0298435 (PMC10889642; doi:10.1371/journal.pone.0298435)
Supplement: Attachment — Submitted filename: Response to reviewers.docx [file pone.0298435.s001.docx]

**Submission PONE-D-23-30870**

**Orthostatic hypotension in stroke/TIA patients: Association with new events and the effect of the NAILED intervention**

Dear Dr. Pinzon

Thank you for comments and the opportunity to resubmit an improved manuscript. Clarifications and additions to the manuscript have been made according to your useful suggestions and we hope that the manuscript now is more comprehensive. We have addressed all the concerns which are detailed below. Changes in the text are also highlighted in the manuscript.

**COMMENT: Please be detail with the method. Is it pre-planned?**

RESPONSE: Measurement of seated and standing blood-pressure was part of the yearly follow-up in the NAILED trial. According to the study-protocol, analysis of standing BP was considered as a secondary outcome and analysis of this as exploratory. An addition to the methods concerning this is now added (page 4, first paragraph): “Standing blood pressure was a prespecified, secondary outcome systematically collected through the course of the study to enable assessment of hypotensive as well as hypertensive reactions since both may be associated with prognosis. Related substudies was, however, not prespecified in further detail. The present study should therefore be considered a post hoc analysis.”

**COMMENT: Do you have data of stroke type and sub-type? Please be detail with the** **radiological findings. Radiological (CT/ MRI) findings?**
RESPONSE: The NAILED trial used a pragmatic approach to include as many patients with stroke or TIA as possible that were able to handle a telephone call. The patients received a TIA or stroke diagnosis according to the treating physician and the protocol did not specify in what way the patient should be investigated and there are not data on stroke sub-type. However, all stroke and TIA patients performed a CT-scan. As described in table 1, of the 814 patients included, 27 (3.3%) had an ICH, 470 (57.7%) an ischemic stroke and 317 (38.9%) a TIA.

**COMMENT: Begin the discussion with the main finding of your study.** 
RESPONSE: The beginning of the discussion is now revised and starts with the main finding (page 8, second paragraph): “In this post-hoc analysis of the NAILED stroke trial, we studied the association between OH and incidence of stroke, AMI, or death in a stroke and TIA population. We found that OH was not associated with any of these outcomes. We also studied the association between group allocation in NAILED and OH and found no indication of increased prevalence of OH when blood pressure was managed according to the NAILED intervention.”

**COMMENT: Please elaborate more your findings with the biological plausibility of the result.**
RESPONSE: We have now added a section on biological plausibility of the result in the discussion (page 9, second paragraph): “After a postural change to standing, blood pools to the lower extremities, leading to decreased venous return and ventricular filling, resulting in reduced cardiac output and lower SBP. Baroreceptors respond to the decreased pressure, triggering an increased sympathetic tone, leading to elevated heart rate and peripheral vascular resistance [23]. If this compensatory mechanism fails, orthostatic hypotension (OH) can occur. Several mechanisms can disrupt this system, including stiff vessels as part of manifest or subclinical vascular disease.”

**COMMENT: Please elaborate more the limitations. Post-hoc data analyses are an issue because they conform to neither the population nor the randomization model of statistical inference. When we discover an apparent difference by way of an unplanned post-hoc analysis, we may have discovered nothing more than simple coincidence.**
RESPONSE: To the discussion on post-hoc analysis we have now added the following text to address this topic (page 10, second paragraph): “…the NAILED trial was not dimensioned based on the outcomes analyzed in the present study. The study may therefore be underpowered, with a risk of a type b error especially in the multivariate analysis. Also, the observational design in this RCT population might be a subject to confounding. We have tried to minimize the risk by adjusting possible confounders but we cannot exclude the possibility of residual confounding.”

**COMMENT: The conclusion should be concise and clear.**

RESPONSE: The conclusion is now updated and hopefully more concise and clearer (page 10, last paragraph): “OH after stroke and TIA was not associated with an increased risk of stroke, AMI, or death. Furthermore, the NAILED stroke trial intervention was not associated with an increased risk of OH during follow-up compared with usual care. Thus, treating BP to a treatment goal of <140/<90 mm Hg in a stroke/TIA population seems safe and fear of OH should not be a reason to deaccelerate BP treatment.”

For your information, I also updated “data collection and outcomes” (page 5, second paragraph) and the update is also highlighted.
