# Supplementary material for: Orthostatic hypotension in stroke/TIA patients: Association with new events and the effect of the NAILED intervention
Source: PLoS One. 2024 Feb 23;19(2):e0298435. doi: 10.1371/journal.pone.0298435 (PMC10889642; doi:10.1371/journal.pone.0298435)
Supplement: Attachment — Submitted filename: Response to reviewers 2.docx [file pone.0298435.s002.docx]

**Submission PONE-D-23-30870**

**Orthostatic hypotension in stroke/TIA patients: Association with new events and the effect of the NAILED intervention**

Dear Dr. Pinzon

We are pleased that you find the manuscript interesting and appreciate the opportunity to resubmit an improved version. Thank you for your valuable comments. We have addressed all concerns detailed below, and changes in the manuscript have been made according to your suggestions, now highlighted in the manuscript. We hope that the manuscript is now more comprehensive.

Reviewer #1

**COMMENT: The paper, having undergone a previous revision, has been meticulously reviewed, with each recommendation from the previous reviewer conscientiously addressed. The overall quality of the paper is commendable, characterized by clear and concise writing. However, it is worth noting that a minor oversight occurred in the manuscript. Specifically, in one instance, the term 'Model 2' was omitted where it should have been included. The sentence in question reads, 'Model 1 was adjusted for age and sex, and Model was adjusted for age, sex, diabetes at baseline.' I suggest that, the correct wording should be 'Model 1 was adjusted for age and sex, and Model 2 was adjusted for age, sex, diabetes at baseline.' This is the only discrepancy identified.**

RESPONSE: Thank you for noticing the missing “2”. This is now adjusted (page 5, last paragraph).

Reviewer #2

**COMMENT: Abstract/Methods: it should be added if this is a prespecified analysis or not**

RESPONSE: The collection of standing blood pressure was prespecified and considered as a secondary outcome while this post-hoc analysis was not prespecified in detail. This is explained in the methods (page 4, first paragraph).

**COMMENT: Methods: Definition of OH should include symptoms. please add a reference for this**

RESPONSE: Consensus documents define OH as a drop in standing systolic blood pressure of at least 20 mmHg or diastolic blood pressure of 10 mmHg. OH, can be symptomatic or asymptomatic. This study included both symptomatic and asymptomatic OH. This is clarified and references are given in the Methods section (page 4, last paragraph and page 5, first paragraph).

**COMMENT: Methods. Subgorup analysis for stroke vs. ematoma should be added**

RESPONSE: We are not sure that we understand exactly what subgroup analysis reviewer 2 would like to be performed. However, with 27 patients with intracerebral hematoma at baseline and 9 outcome-events of stroke that was not ischemic, there will be too few cases for any meaningful subgroup analysis.

**COMMENT: Methods. It should be added how variables were choosen**

RESPONSE: Baseline variables were collected during the index hospitalization and at the 1-month follow-up according to the NAILED study protocol. A reference for this is included in the manuscript and for clarity now also added after the description of baseline data (page 5, second paragraph): “[19]”. A reference for the process and definitions of new cardiovascular events is also included in the manuscript.

**COMMENT: Methods: performance of multivariatre model should be added**

RESPONSE: The multivariable models are described in the statistical analysis section (page 5, last paragraph) and the results are presented in table 3.

**COMMENT: Adjiducagtion of OH: it should be added when was performed and at which timeline**

RESPONSE: Measurement of seated and standing BP was performed 1 month after discharge of index event and then at the yearly visits in the NAILED trial. This is explained in the manuscript (page 4, last paragraph).
